# Commentary on Zacher *et al*.: How cocaine memories may drive craving and drug instrumentalization

**DOI:** 10.1111/add.70532

**Published:** 2026-06-25

**Authors:** Christian P. Müller

**Affiliations:** ^1^ Department of Psychiatry and Psychotherapy University Clinic, Friedrich‐Alexander‐University of Erlangen‐Nüremberg Erlangen Germany; ^2^ Institute of Psychopharmacology, Central Institute of Mental Health, Medical Faculty Mannheim Heidelberg University Mannheim Germany

**Keywords:** addiction, cocaine, craving, drug instrumentalization, intrusions, memory

*In drug addiction, the relationship between drug‐related memory intrusions, craving and drug taking is unclear. A new study suggests a more frequent drug‐memory retrieval and assessment of cocaine instrumentalisation than the urge to really take the drug. Most cocaine‐related memories seem not to lead to craving and drug taking*.

Many episodes of drug use are not primarily motivated by the pursuit of euphoria or happiness. Drug use is much more systematic and may serve a number of different goals. Therefore, a drug is used to change a given mental state into a desired state, in which a goal‐directed and rewarded behaviour is performed more efficiently. Such functional drug use can be called drug instrumentalisation. For cocaine, the goals are often to boost social behaviours, sexual behaviours or work performance in creative jobs [1, 2]. Thereby, drug choice is especially selective for time, personal state and is context dependent. Instrumental motives are likely to be a prominent characteristic for individuals who are not dependent on cocaine, but elements of this pattern are likely to remain prominent if cocaine use disorder (CUD) is established [3, 4, 5].

Importantly, drug instrumentalisation is a learned behaviour. In fact, not only the amount and way of consumption need to be learned, but also the instrumental use of the drug itself. To achieve this, numerous memory systems are recruited [1, 6]. Only systematic retrieval of the information of when to take the drug, in what amount and for what kind of behaviour, together with the expected outcome, is likely to support a systematic pattern of drug use. This suggests a clear differentiation of behavioural content and expectations that may benefit from cocaine effects from those that do not. In these respects, memory intrusions, whether cue‐induced or spontaneously emerging, may play an important role in decisions about cocaine use.

Craving appears as a subjectively perceived urge to seek and take the drug. This may be seen as the wish to change the current mental state into a previously learned and remembered drug‐induced state. Craving may result in drug seeking and taking behaviour, but for cocaine this is not always the case [7]. As it is usually reported by individuals who use cocaine, craving may not involve specific memories for drug instrumentalisation [8, 9].

Currently, the relationship between drug‐related memory intrusions and craving is unclear. The study by Zacher and colleagues [10] addressed this by measuring the nature of its relationship in individuals with CUD. Thereby, the ecological momentary assessment (EMA) provided an unprecedented temporal resolution that is very welcome and virtually required to make valid conclusions [11, 12]. They found that drug‐related memory intrusions often occurred independently of the craving. This may, on the one hand, suggest that there is a more frequent drug‐memory retrieval and assessment of the possibility of cocaine instrumentalisation in an everyday context, than the actual urge to really do it. Therefore, most drug‐related memories seem not to lead to craving and drug taking. On the other hand, the authors report that when intrusions become more vivid and intense, they may co‐occur most closely with craving. From these findings, one may conclude that there is a graded relationship between the intensity of memory retrieval and experience and the likelihood of craving. Only when memory retrieval becomes intense (although the mechanisms underlying this remain unclear) and a threshold is reached, motivational centres in the brain become sufficiently activated and produce the subjective feeling of craving and subsequent drug seeking and taking behaviour.

At this point it may become interesting to further elaborate the nature of the memory intrusions and craving in the future to see how close the content of the intrusions relates to, for example, situation‐ or task‐specific craving and consumption. Such information may be highly relevant for behavioural therapies that seek to interfere with drug‐related memories and the cognitive processes through which they may contribute to craving and cocaine use.

## AUTHOR CONTRIBUTIONS

**Christian P. Müller:** Writing—original draft; writing—review and editing.

## DECLARATION OF INTERESTS

The author is editor‐in‐chief of the journal, *Behavioural Brain Research*. No financial or other relevant links to companies with an interest in the topic of this article.
